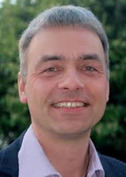# Chiral Triptycenes: Concepts, Progress and Prospects

**DOI:** 10.1002/chem.202005317

**Published:** 2021-02-26

**Authors:** Md. Nasim Khan, Thomas Wirth

**Affiliations:** ^1^ School of Chemistry Cardiff University Park Place, Main Building Cardiff CF10 3AT UK

**Keywords:** absolute configuration, catalysis, chiral advanced materials, chiral triptycene, circular polarized luminescence

## Abstract

Triptycenes have been established as unique scaffolds because of their backbone π‐structure with a propeller‐like shape and saddle‐like cavities. They are some of the key organic molecules that have been extensively studied in polymer chemistry, in supramolecular chemistry and in material science. Triptycenes become chiral molecules when substituents are unsymmetrically attached in at least two of their different aromatic rings. This Minireview highlights the chirality of triptycenes from basics to an advanced stage for the development of functional molecules.

## Graphical abstract

**From basics to advanced**: Chirality in triptycenes due to atropisomerism and stereocenters have been reviewed and the routes to obtain these chiral triptycenes have been highlighted. This Minireview describes the initial history of developments of chiral triptycenes and its comeback to important applications in advanced materials and catalysis after a gap of almost 30 years.

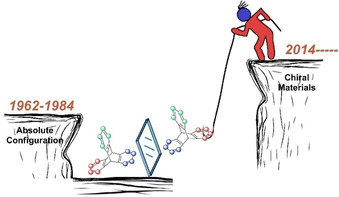

## Introduction

**Figure 1:**
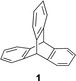
Triptycene **1**.

Triptycene is the simplest member of the iptycene family with a bridged bicyclooctatriene core structure (Figure 1). With its *D*_3*h*_ symmetry and 120° angles between the three aromatic rings in a paddle‐wheel rigid structure, the molecule provides a large free volume around the aromatic blades.^[1]^ Because of these unique structural features, triptycenes have been incorporated as molecular scaffolds and extensively studied in various areas such as polymer chemistry, material chemistry, molecular machines, nanosized molecular cage, molecular balances, medicinal chemistry, peptide chemistry, molecular assembly, host‐guest chemistry and also catalysis.^[2]^ 1,9‐Disubstituted triptycenes have been valuable compounds for the investigation of π‐interactions in aromatic systems.^[3]^ The triptycene scaffold provides adequate reactive positions including sp^3^ and sp^2^ sites to install reactive handles and extensions of the π‐scaffold in designing triptycene derivatives. Although the sp^3^ positions in triptycenes are difficult to functionalize compared to normal benzylic positions,^[4]^ some strategies have been applied to make π‐extended structures.^[5]^ A large number of triptycene derivatives with functional groups at different positions has been synthesized, but regiospecific functionalizations of triptycenes at the *ortho*‐positions next to the sp^3^ carbons are limited. Mostly achiral triptycenes have been explored in areas of synthesis and material applications although the first synthesis of chiral triptycenes was reported in 1962.^[6]^ Recent results of chiral triptycenes has awakened them from a three decades sleep to become an important player in modern chemicals science. From synthesis to applications, achiral triptycenes are well documented but chiral triptycenes have not been reviewed and only very recently a survey has been published.^[7]^ This Minireview covers the concept and highlights the development and applications of chiral triptycenes. It also includes clarifications of previously studied chiral triptycenes wherever available and shines a light on future prospects.

## Chirality in Triptycenes

**Figure 2:**
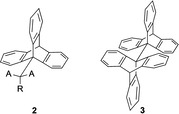
Structure for 9‐substituted triptycenes **2** and 9,9′‐bitriptycyl **3**.

**Scheme 1:**
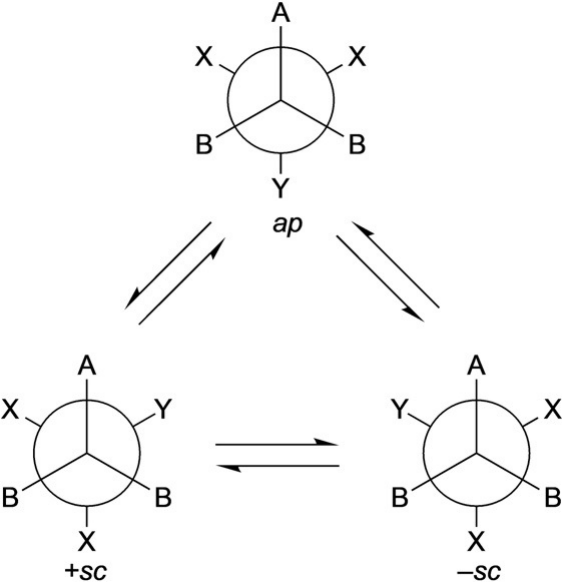
Rotational circuit of AB_2_C‐CX_2_Y.

**Scheme 2:**
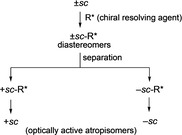
Flow diagram for the isolation of atropisomers +*sc* and −*sc*.

Chirality in triptycenes have been studied in two different areas of stereoisomerism. Atropisomers have been investigated as well as triptycenes with stereocenters having defined configuration. In atropisomers the chiroptical properties are correlated with a pair of enantiomeric conformations.^[8]^ For this, optically active and inactive rotational isomers of triptycene have been isolated and characterized. It is known that in many cases of organic compounds, it has been difficult to isolate optically active conformational isomers to study their chiroptical properties at room temperature. Even if isolated, they rapidly interchange and racemization occurs at room temperature. To understand the chemistry of atropisomers, triptycene derivatives of types **2** and **3** (Figure 2) have been explored because substituents at the 9‐position or at the *peri* position (*ortho*‐position in aromatic ring closer to substituents at 9‐position) can provide a high barrier to rotation and lead to stable conformational isomers at room temperature. Triptycene derivatives of the general formula AB_2_C‐CX_2_Y can undergo a rotational circuit as shown in Scheme 1. Here, the *ap*‐conformer has a plane of symmetry and so it would be optically inactive. Because of the internal rotation, this compound may take the conformations as either +*sc* or −*sc* form. The −*sc* form and +*sc* form are mirror images and have *C*_1_ symmetry. Both isomers +*sc* and −*sc* would be optically active if isolated. A common strategy used to isolate the optically active conformers are shown in Scheme 2. Racemic ±*sc* was treated with a chiral resolving agent and then each diastereomers were isolated. Finally, the removal of the chiral resolving agent produces the optically active conformers +*sc* and −*sc*, respectively.

**Figure 3:**
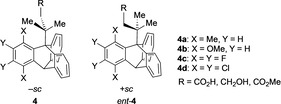
Optically active triptycene atropisomers **4**.^[8c]^

As an example, triptycene derivatives **4** and *ent*‐**4** (Figure 3) of the general formula AB_2_C‐CX_2_Y have been synthesized, isolated and characterized as optically active atropisomers.^[8c]^ Similarly, the conformations of 9,9′‐bitriptycyl derivatives with the general formula XCH_2_‐CH_2_X and XYZC‐CXYZ have been studied. Very recently, a new strategy has been explored using an enantioenriched aryne atropisomers for the synthesis of chiral triptycenes.^[8d]^

**Figure 4:**
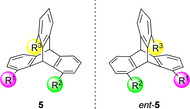
Chiral triptycenes **5** (R^1^≠R^2^≠R^3^).

Configurationally chiral triptycenes can be prepared when at least two of the aromatic rings are unsymmetrically substituted. Structure **5** (Figure 4) shows a 1,8,13‐trisubstituted triptycene and is chiral when R^1^ ≠ R^2^ ≠ R^3^. Compounds **5** and *ent*‐**5** are non‐superimposable mirror images and therefore enantiomers.

Optically active chiral triptycenes have also been prepared by attaching enantiomerically pure molecule to the triptycene unit.^[9]^ The chiral auxiliary (*S*)‐3,7‐dimethyloctyl bromide has been connected to a triptycene moiety to obtain a chiral polymer.^[10a]^ Similarly, (*R*)‐(+)‐1,1’‐bi‐2‐naphthol has been used in the synthesis of chiral triptycene‐based receptors^[10b]^ and (1*S*,2*S*)‐1,2‐diphenyl‐1,2‐diaminoethane for generating chiral triptycene‐based *N*‐heterocyclic carbene ligands.^[10c]^

## Progress

### Chiral triptycenes: Early 90s development

**Figure 5:**
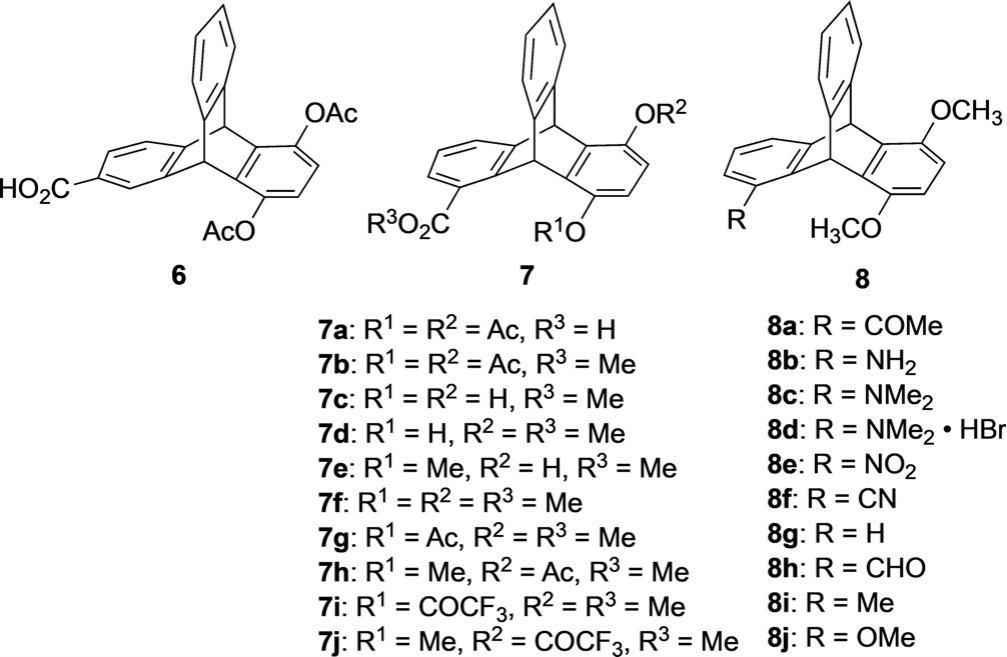
Structures of 5,8‐diacetoxy‐9,10‐dihydro‐9,10‐[1,2]benzenoanthracene‐2‐carboxylic acid **6** ^[6]^ and chiral triptycene derivatives **7** ^[11]^ and **8**.^[13]^

The first synthesis of chiral triptycenes was reported by Nakagawa and co‐workers in 1962 with the compound 5,8‐diacetoxy‐9,10‐dihydro‐9,10‐[1,2]benzenoanthracene‐1‐carboxylic acid as a trisubstituted triptycene.^[6]^ Nakagawa and his group wanted to study the relationship between the optical activity and differently substituted functional groups in a molecule, for which they selected the chiral triptycene molecule. The reason for their choice of chiral triptycenes was the rigid and fixed geometry of these derivatives. Therefore, triptycene can exclude the influence of the complicated effects that generally arise in flexible molecule while studying the optical activity in relation to substituted functional groups. Initially, compound **6** (Figure 5)^[6]^ was prepared in a stepwise manner but the resolution of *rac*‐**6** using naturally occurring chiral bases was unsuccessful. They prepared **7** instead and resolved the enantiomers using brucine tetrahydrate. Pleasingly, with this strategy several optically active triptycenes based on the structure **7** were synthesized (Figure 5).^[11]^ It was expected that all these derivatives should have the same configuration as that of the parent triptycene **7 a**, because the rigidity of the triptycene avoids any racemization or Walden inversion during the course of transformation. From the study of the rotatory dispersion (RD) curves it was observed that the sign of plain curves of **7 g** and **7 i** were positive whereas **7 h** and **7 j** showed negative plain curve despite having the same configurations as **7 g** and **7 i**. From the analysis it was concluded that the determining factor for the change in the sign of the RD‐curve was guided by the direction of the electronic polarization.

**Figure 6:**
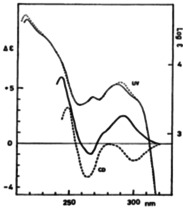
UV and CD spectra of **7 g** (—) and **7 h** (‐ ‐ ‐ ‐) in dioxane. Reproduced/Adapted from ref. [12] with permission from The Chemical Society of Japan.

Further CD analysis (Figure 6)^[12]^ of the diastereomeric compounds **7 g** and **7 h** shows the sign of the longest wavelength Cotton effect is reversed as suggested by the ORD data.^[11]^ This indicates that the center of the transition dipole of the ^1^B_2u_ state in the hydroquinone ring is displaced in the opposite direction according to the inversion of the position of electron attractive substituent. However, the CD spectral pattern at shorter wavelength region suggests that the effect is restricted to the ^1^B_2u_ state.

In the process of their studies Nakagawa et al. synthesized a series of functionalized optically active **8** (Figure 5)^[13]^ from **7 a** following different functional group transformation steps and determined the absolute configuration (1*R*,6*S*) for the derivative **8 d** using X‐ray crystallographic analysis. It revealed that all the synthesized compounds in this series have the same absolute configuration and shows dextro optical rotation regardless of the substituent in the 1‐position.

In a similar way Nakagawa et al. synthesized other chiral triptycenes, determined their absolute configuration by correlation methods and studied their UV and CD properties.^[14]^

The absolute configuration of chiral compounds can also be determined by using electronic circular dichroism exciton chirality method in a non‐empirical way without using any known reference compound with absolute configuration.^[15]^ In this direction Harada et al. studied and determined the absolute configuration of benzo‐extended chiral triptycene derivatives.^[16]^ This was the first report of an unambiguous and nonempirical way to determine the absolute configuration of chiral triptycenes applying the CD exciton chirality method in support with quantum mechanical calculations.

### Chiral triptycenes: Post‐90s development

**Scheme 3:**
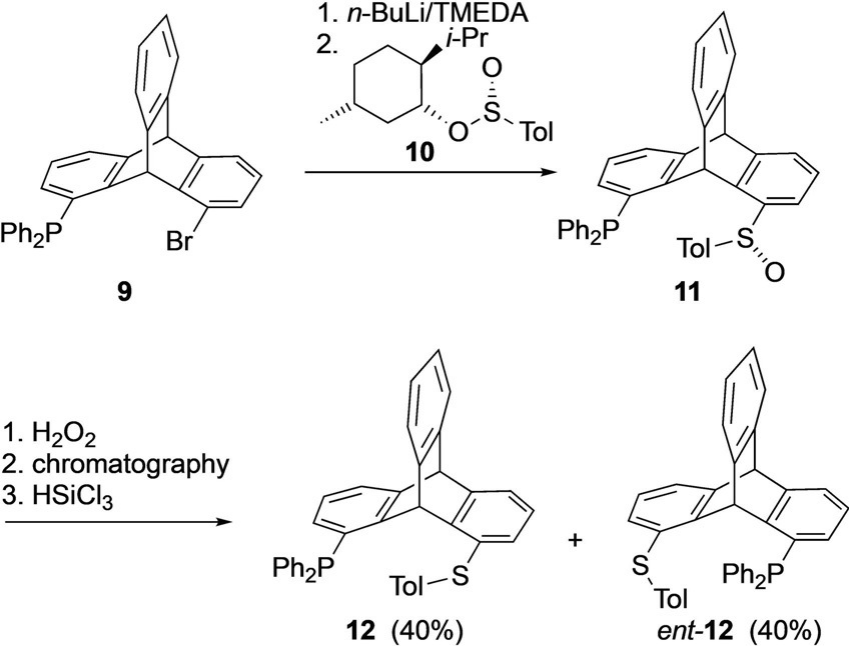
Synthesis of chiral triptycene based phosphine ligands.^[17]^ Tol: 4‐Me‐C_6_H_4_.

After the synthesis and CD exciton chirality studies by Harada et al. of triptycenes embedded in an anthracene chromophore, a huge gap in the research of chiral triptycenes can be recognized. After an interval of 30 years, Gelman and co‐workers reported the synthesis of the chiral monophosphine ligand **12** and *ent*‐**12** (Scheme 3).^[17]^ The first step involves the preparation of a *rac*‐1‐bromo‐8‐diphenylphosphinotriptycene **9** from 1,8‐dibromotriptycene. *Rac*‐**9** was treated with *n*BuLi followed by quenching with (1*R*,2*S*,5*R*)‐(−)‐menthyl‐(*S*)‐*p*‐toluenesulfinate **10** producing a mixture of diastereomers **11** which were then separated by column chromatography. The absolute configuration was assigned with the help of a single crystal X‐ray analysis. Isolated yields for **11** were low because of their sensitivity towards oxygen. Therefore, the diastereomeric mixture **11** was first oxidized with hydrogen peroxide to make the corresponding phosphine oxides which were then isolated chromatographically and finally reduced together with the sulfoxides using HSiCl_3_ to obtain **12** and *ent*‐**12** as chiral ligands.

**Scheme 4:**
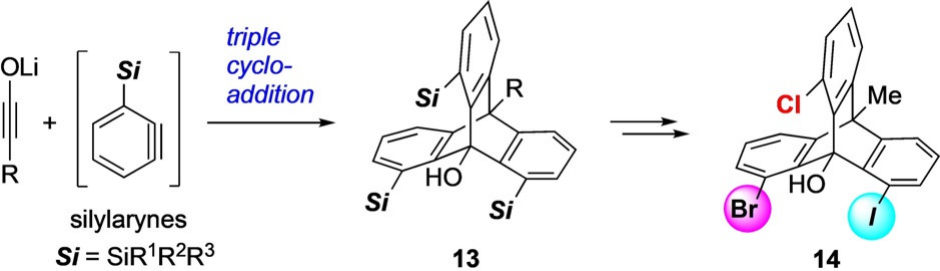
Synthesis of bromo‐chloro‐iodo‐substituted chiral triptycene **14**.^[18]^

Shindo et al. reported a synthetic route for the synthesis of trisilyltriptycenes **13** in a highly regioselective manner by triple addition of ynolates and 3‐silylbenzynes.^[18]^ The functionalized intermediate **13** was halogenated stepwise to obtain bromo‐chloro‐iodo‐substituted chiral triptycene **14** (Scheme 4). Compound **13** presents a very useful platform to make a variety of 1,8,13‐trisubstituted chiral triptycenes.

**Scheme 5:**
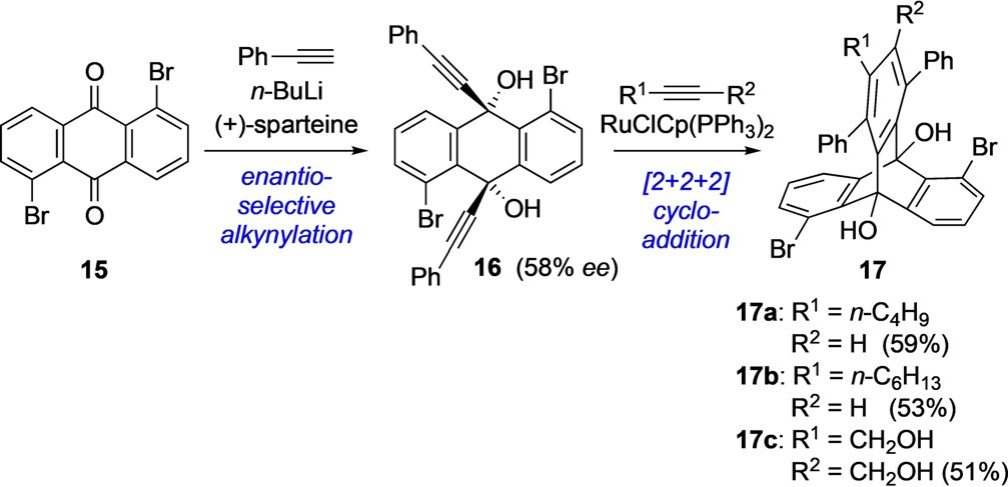
Synthesis of chiral 1,5‐dibromo‐triptycenes **17**.^[19b]^

A new strategy was applied for the synthesis of **17** from **15** in an achiral protocol.^[19a]^ Its enantioselective synthesis was reported by Shibata et al. and is considered to be the first report to obtain an optically active triptycene.^[19b]^ The initial step involves an enantioselective alkynylation of 1,5‐dibromoanthracene‐9,10‐dione **15** catalyzed by (+)‐sparteine. In the second step, intermediate **16** underwent a rhodium‐catalyzed [2+2+2] cycloaddition with alkynes to obtain the desired chiral 1,5‐dibromo‐triptycenes **17** (Scheme 5). Product **17 c** was used for the synthesis of functionalized chiral diaryl‐substituted triptycenes applying Suzuki coupling reactions.

**Scheme 6:**
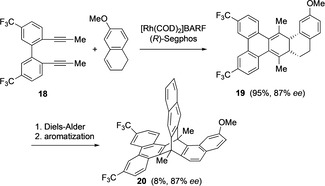
Synthesis of distorted π‐extended chiral triptycene **20**.^[20]^

A second enantioselective synthesis of chiral triptycene based on rhodium‐catalyzed [2+2+2] cycloaddition was reported by Tanaka et al. (Scheme 6).^[20]^ The key step involves a Rh^I^‐catalyzed enantioselective addition of biphenyl‐linked diyne **18** and 1,2‐dihydronaphthalene in the presence of (*R*)‐Segphos resulting in the chiral polycyclic cyclohexadiene **19** in excellent yield with 87 % *ee*. The next step involved a diastereoselective Diels–Alder reaction between **19** and 1,4‐naphthoquinone. Upon reduction and aromatization, a distorted π‐extended chiral triptycene **20** was obtained without racemization. Compound **20** was used further to explore other chiral triptycene derivatives.

**Scheme 7:**
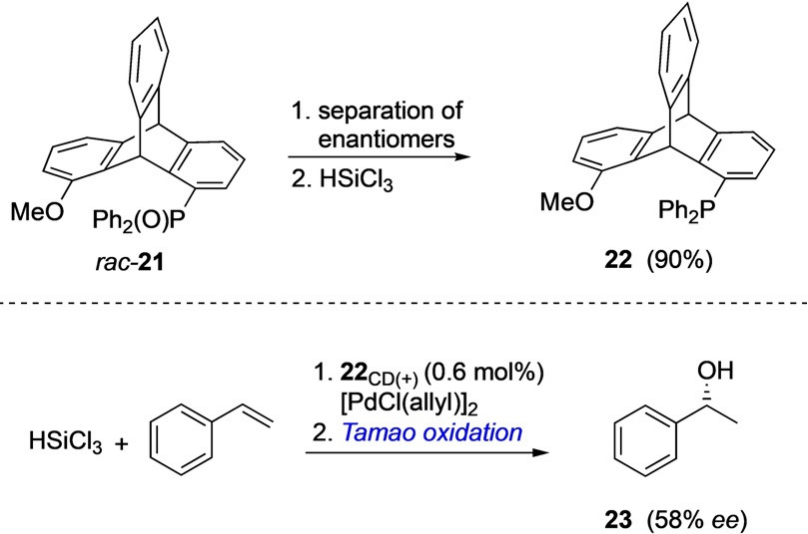
Synthesis of chiral monophosphine ligand **22** and its use in asymmetric hydrosilylation.^[21]^

Chiral triptycenes as catalysts in asymmetric synthesis were explored for the first time by Leung et al. in 2017. Chiral monophosphine ligand **22** was synthesized in a stepwise manner starting from 1,8‐dihydroxytriptycene via compound **21** (Scheme 7, top).^[21]^ Optical resolution of **22** was unsuccessful, therefore the enantiomers of its precursor **21** were separated using chiral HPLC and were subjected to UV and circular dichroism (CD) studies. The isolated isomers of **21** were reduced to **22** using HSiCl_3_. The monophosphine ligand **22** was used in Pd‐catalyzed Suzuki–Miyaura cross‐couplings where no asymmetric induction was observed, but in the asymmetric hydrosilylation the reduced product **23** was obtained with 58 % *ee* (Scheme 7, bottom).

**Scheme 8:**
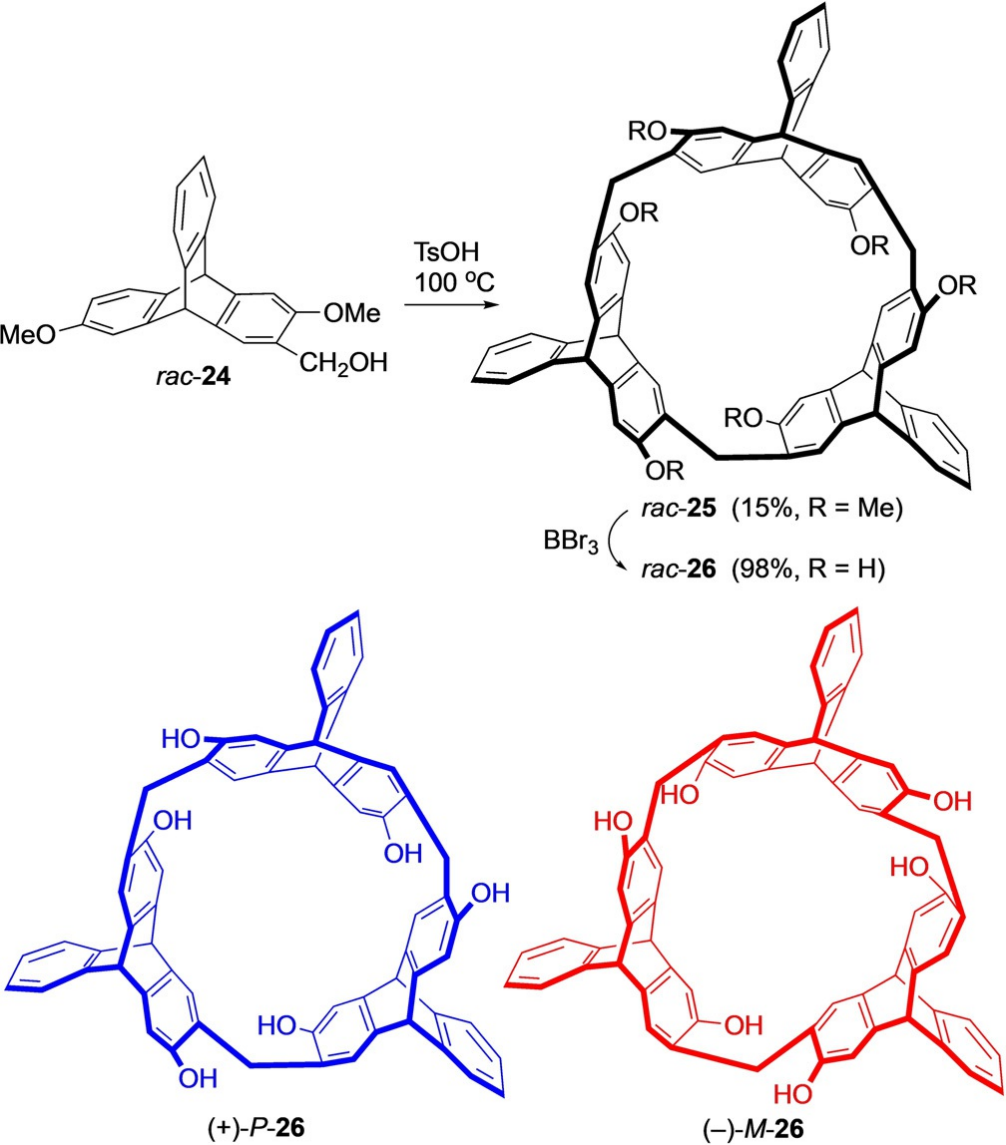
Synthesis of chiral 2,6‐helix[6]arene **26**.^[24]^

The recent advancement of chemical science to create high quality materials for various applications has also involved the search for new and useful structures. Triptycenes possess such features due to their special arrangement and have therefore been studied in polymer science, supramolecular chemistry and materials chemistry, but only as achiral compounds. Materials with chiroptical properties have been investigated for applications in advanced technologies such as three‐dimensional displays, quantum computing and teleportation as they show electronic circular dichroism (ECD) and circularly‐polarized luminescence (CPL).^[22]^ Molecules have to be designed that they should either have an helical molecular geometry or possess chiral supramolecular assemblies.^[23]^ Chiral triptycenes provide such building blocks suitable for the synthesis of chiroptical materials. Interesting results obtained from the work based on chiral triptycenes have attracted attention, but the examples are scant. Based on the chiral triptycene building block 2,6‐dihydroxytriptycene and its derivatives chiral macrocyclic arenes have been synthesized. Chen et al. reported the synthesis of a chiral 2,6‐helix[6]arene **26** ^[24]^ where chirality is generated by using 2,6‐dimethoxy‐3‐hydroxymethyltriptycene **24** as shown in Scheme 8. *Rac*‐**26** was obtained after the cleavage of the methoxy groups and resolved into the two enantiomers *P*‐**26** and *M*‐**26** using (+)‐camphorsulfonyl chloride. The determination of the absolute configurations was performed by X‐ray diffraction and CD analysis. This methodology was extended further to the synthesis of a library of macrocycles that have been used in the studies for potential applications in chiral recognition, stimuli‐responsive host‐guest complexation and molecular machines.[^2j^, ^25^]

**Figure 7:**
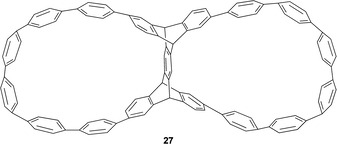
Structure of nanohoop molecule **27**.^[26]^

Cycloparaphenylene are referred to as ‘carbon nanohoops’ and are strained carbon nanotube structures that show interesting size‐dependent optoelectronic and host‐guest properties. Xu et al. have reported a new type of chiral dual nanohoop molecule **27** (Figure 7) and studied their chiroptical properties by CD and CPL spectroscopy.^[26]^ The key step involved in the synthesis was a ring expansion through dianthracene cycloreversion followed by a transannular [4+2] cycloaddition in a 64‐membered macrocycle. The enantiomers of **27** were resolved by chiral HPLC.

**Scheme 9:**
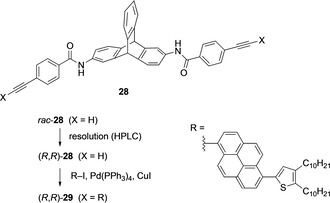
Synthesis of triptycene‐pyrene hybrid molecule **29**.^[27]^

**Figure 8:**
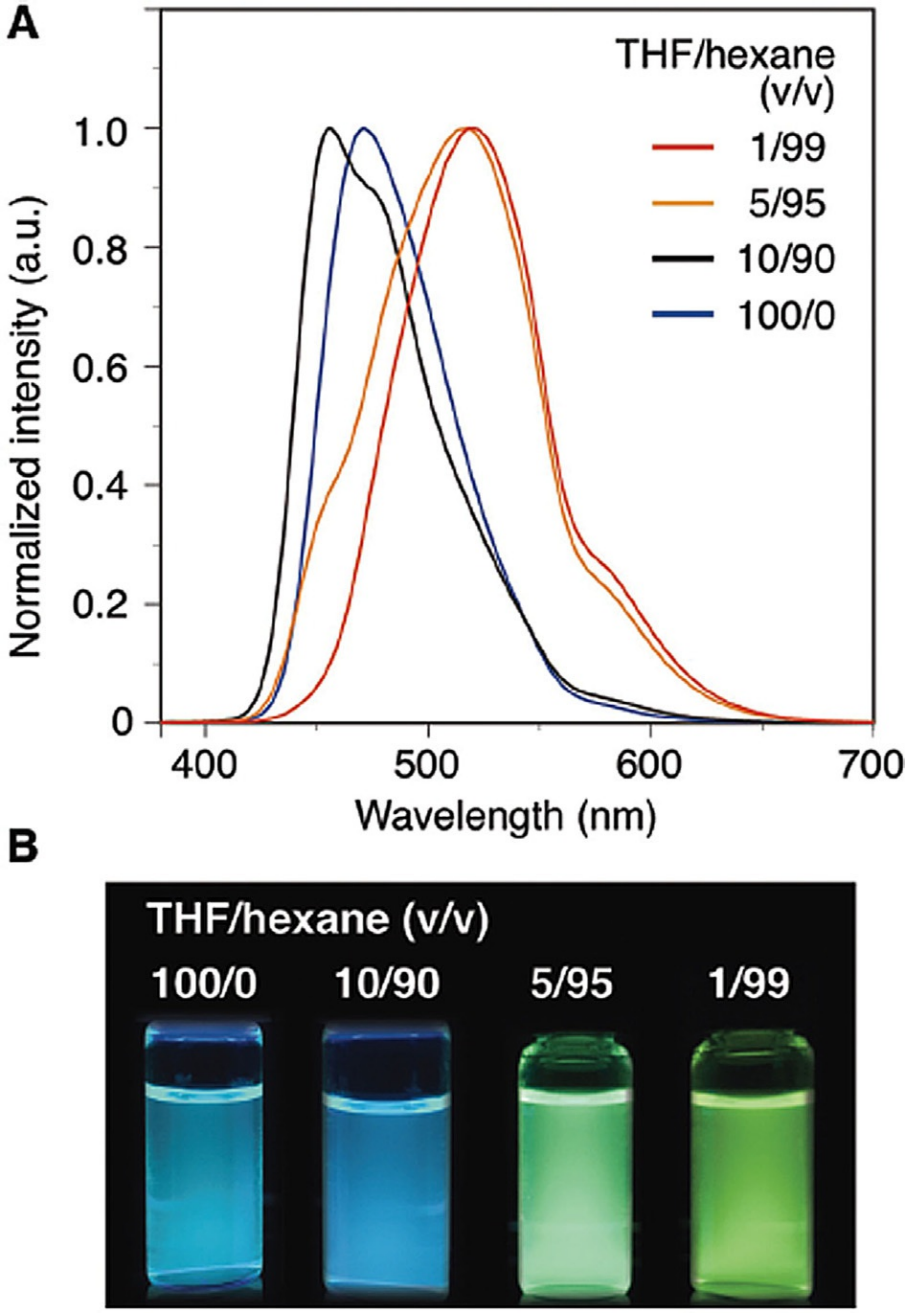
A: Fluorescence emission spectra of (*R*,*R*)‐**29** (*λ*_ex_=365 nm) in THF/hexane (100/0–1/99, v/v) at room temperature. [**29**]=1.0×10^−5^ M^.^ B: Photograph of the corresponding solutions under UV irradiation (365 nm). Reproduced/Adapted from ref. [27] with permission from The Royal Society of Chemistry.

**Figure 9:**
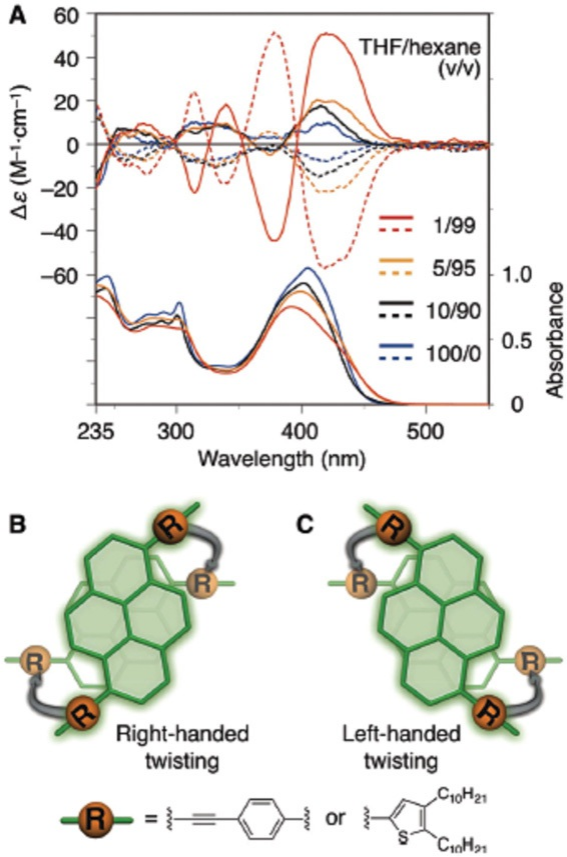
A: CD and absorption spectra of (*R*,*R*)‐**29** (solid line) and (*S*,*S*)**‐29** (dashed line) in THF/hexane (100/0–1/99, v/v) at 25 °C. [**29**]=1.0×10^−5^ M. B: Schematic illustration of right‐handed twisted structures of the pyrene‐based π‐conjugated units. **C**: Left‐handed structure. Reproduced/Adapted from ref. [27] with permission from The Royal Society of Chemistry.

In parallel with the development of chiral macrocycles and ligands, chiral triptycene scaffolds have also been explored in the synthesis of chiral functional materials. The scarce examples show that chiral triptycenes have the potential to provide a platform for making advanced functional materials. Swager and co‐workers have reported the synthesis and potential of chiral triptycenes as luminescence material in 2017. As supramolecular chirality is induced in hydrogen‐bonded aggregates, the synthesized chiral molecules emitted left‐ or right‐handed circularly polarized light (CPL) after irradiation with UV light. A limiting factor is that these small molecules can show CPL only when they are in an aggregated state. This special feature limits them in making practical applications of materials, as the material should be free from any influence of external effects such as temperature, solvent and concentration. The synthesized triptycene‐pyrene hybrid system is shown in Scheme 9.^[27]^ The first step in this synthetic route involves the amide formation of *rac*‐2,6‐diaminotriptycene and 4‐ethynylbenzoic acid. The obtained compound *rac*‐**28** was subjected to preparative chiral HPLC. The enantiomers (*R*,*R*)‐**28** (and (*S*,*S*)‐**28**) were isolated and the absolute configurations determined by single crystal X‐ray analysis. The subsequent Sonogashira–Hagihara cross‐coupling reaction with pyrenyl compound R‐I led to optically pure (*R*,*R*)‐**29** [and (*S*,*S*)‐**29**]. It was observed that the fluorescence emission and the chiroptical properties of **29** were dependent on the solvent and its concentration. Compound **29** in the solvent mixture of THF and hexane showed a red shift of emission from 470 nm (blue emission) to 520 nm (green emission) when the volume of hexane was increased (Figure 8). The addition of hexane resulted in aggregation induced excimer formation between the pyrenyl units. CD and absorption spectra for (*R*,*R*)‐**29** and (*S*,*S*)‐**29** are shown in Figure 9. When the solvent combination was changed from THF to THF/hexane, a variation in the CD spectra was observed with a hypsochromic effect (absorption spectrum). The CD signals were weak in THF but increased fivefold in THF/hexane. The chiral hydrogen bonded aggregates had a preferred handed twist of stacked pyrene units; (*R*,*R*)‐**29** stacked in a clockwise orientation (Figure 9, B and C). The average size of the aggregates was determined with dynamic light scattering (DLS) and found to be >200 nm in THF/hexane (1/99 v/v %). In the same solvent the aggregates showed a circularly polarized luminescence (CPL) signal reaching dissymmetry factors as high as 1.5×10^−3^.

**Figure 10:**
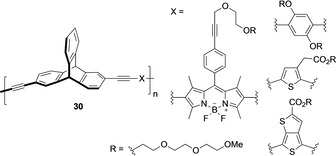
Optically active polymers **30**.^[29]^

The CPL emission was observed only in the aggregate form so there are limited practical applications. From a practical point of view Swager and co‐workers realized that it was necessary to make new types of materials that can show CPL activities independently without being affected by other environmental factors.^[28]^ In the search for such chiral materials where aggregation could avoided, chiral triptycene building blocks were explored and a series of triptycene‐based optically active polymers **30** was synthesized (Figure 10).^[29]^ Comparing CD spectra of the polymers with the monomer and the computationally designed model compounds revealed that the chiral triptycene unit was repeated in the polymer backbone but the polymer structure was not helical. Therefore, the CPL emission properties were independent and not affected by other environmental factors. The glum value (dissymmetry factor) of these polymers, obtained from CPL studies, was almost constant and independent of the included chromophore. It means that the wavelength of the fluorescence can be modified by changing the achiral co‐monomer without effecting CPL properties.

**Scheme 10:**
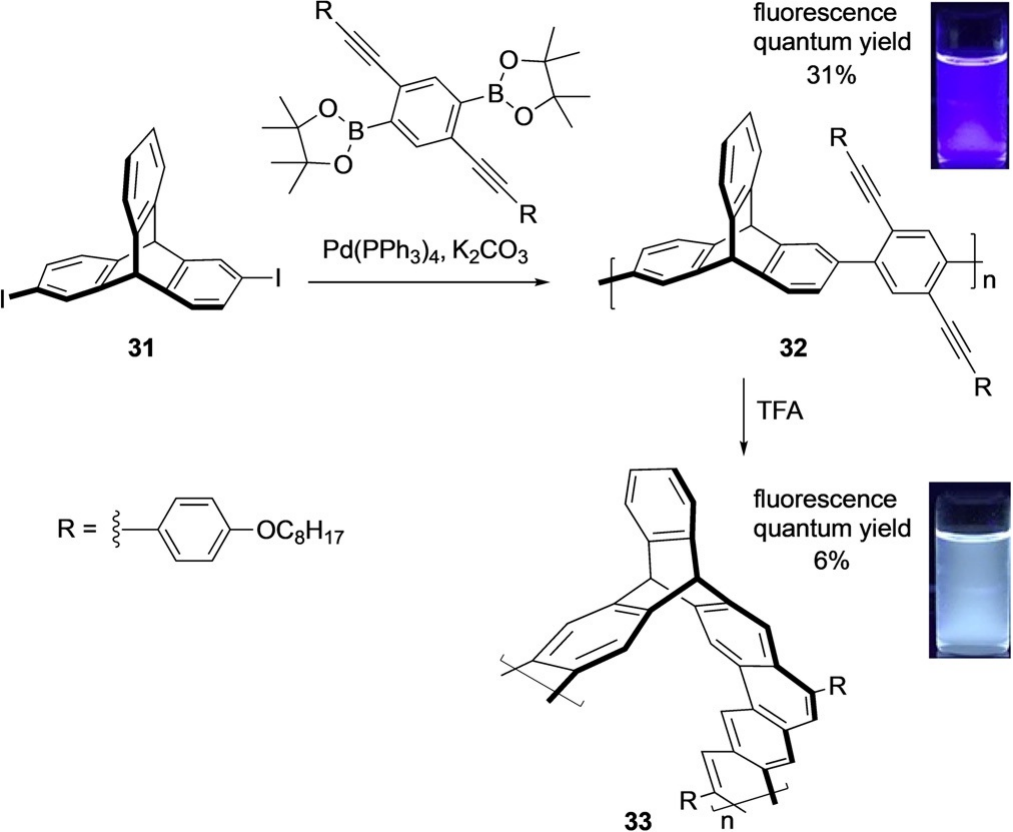
Synthesis of optically active single‐handed helical ladder polymers **33**.^[30]^

Expanding the synthetic scope, optically active single‐handed helical ladder polymers employing intramolecular cyclisation of rigid chiral framework of triptycenes were synthesized.^[30]^ It is noteworthy to mention that the aromatic electrophilic substitution proceeded in a highly regioselective manner at positions 3 and 7 in the triptycene moiety (Scheme 10) in quantitative yield. The perfect regioselectivity was attained because of the steric repulsion between the bridgehead proton and the 4‐alkoxyphenyl substituent. The first step reaction involves the Suzuki–Miyaura coupling copolymerization of enantiomerically pure 2,6‐diiodotriptycene **31** with diboronic acid bis(pinacol) ester containing two 4‐alkoxyphenylethynyl substituents. Polymer **32** with a random coil structure formed **33** on treatment with TFA, a one‐handed helical structure. The number‐average molecular mass, M_n_(SEC) value was estimated 1.05×10^4^ g mol^−1^ for 32 and 0.80×10^4^ g mol^−1^ for **33**, respectively. It was assumed that cyclization lowered the radius of gyration in **32** and so lowered the M_n_(SEC) value with a restricted structural freedom in **33**. When the backbone conformation with a random coil in **32** was changed to a rigid ladder structure in **33**, a change in the photoluminescence (PL) was observed with a 38 nm red shift due to the higher degree of electronic delocalization. A bluish white emission in **33** was observed because of a broad PL band in the region of 470–600 nm. The UV spectrum of **33** were independent of temperature because of the rigid helical ladder structure and showed an intense CD signal compared to **32**. Polymer **33** was used as a chiral stationary phase for high‐performance liquid chromatography (HPLC) with resolution ability.

**Scheme 11:**
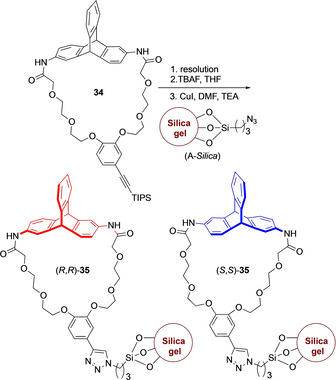
Synthesis of triptycene based chiral stationary HPLC phases.^[31]^

In the development of chiral stationary phases for HPLC, Swager and co‐workers had already explored the use of chiral triptycene synthon. The distorted cyclic structure **34** (Scheme 11) was synthesized from *rac*‐2,6‐diaminotriptycene.^[31]^ Both enantiomers were separated using preparative HPLC on a chiral column. Absolute configurations of **34** were assigned by comparing CD spectra with optically active 2,6‐diaminotriptycene. After silyl group removal, the subsequent Huisgen 1,3‐dipolar cycloaddition with an azide functionalized silica gel formed a chiral stationary phase **35** (CSP) which was utilized efficiently for the resolution of axially chiral biphenyl compounds.

**Figure 11:**
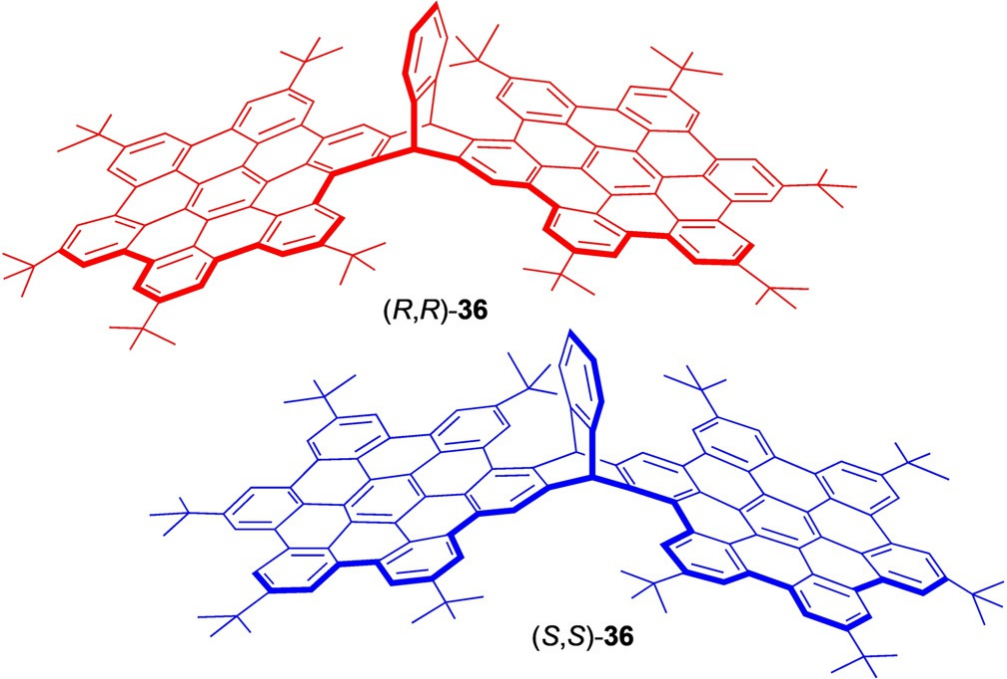
Optically active triptycene **36** with hexa‐peri‐hexabenzocoronene units.^[33]^

An achiral three dimensional (3D) nanographene with hexa‐peri‐hexabenzocoronene incorporating a triptycene unit has been explored as a fluorescent agent for in vitro and in vivo fluorescence imaging.^[32]^ Wada et al. reported a method for the synthesis and isolation of **36** as an asymmetric 3D nanographene framework (Figure 11).^[33]^ In search for a CPL‐active material, Ikai and his group have made this new asymmetric 3D nanographene bearing triptycene scaffolds with a maximum |g_lum_| value of 1.0×10^−3^ matching those reported for conjugated chiral organic materials.^[34]^ In addition, a conglomerate crystallization was observed during crystallization of a racemic mixture of **36**. The conglomerate allowed to generate the right‐ and left‐handed circular polarized light without using any special instruments. This interesting property could provide a convenient approach for the synthesis of chiroptical materials without enantiomeric resolution.

## Prospective

**Figure 12:**
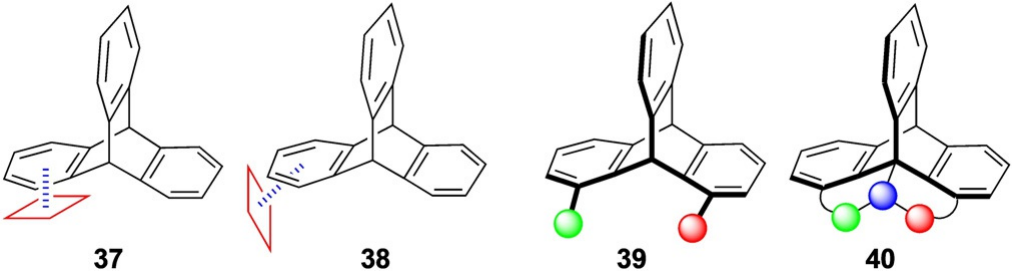
Interactions with triptycenes and chiral structures.

Triptycenes possess important structural features such as a π‐systems and saddle‐like cavities. Due to these properties, they have been extensively explored in many areas of chemistry with useful applications. Similar possibilities remain with chiral triptycenes. The outcome may be amazing for chiral triptycenes if used in the same way as achiral triptycenes have been explored. Recently, some developments based on chiral triptycenes showed interesting properties such as stimuli‐responsive host‐guest complexation, chiroptical properties and as a chiral stationary phases in HPLC. These results show that chiral triptycenes have high potential in future developments. 1,9‐Disubstituted triptycenes are valuable tools in studying π‐interactions in aromatic systems.^[3]^ Non‐covalent π‐interactions have opportunities in catalyst design.^[35]^ Depending on non‐covalent interactions, different approaches for substrates are possible towards a triptycene moiety in a side‐on (**37**) or and end‐on approach (**38**) (Figure 12). The feature of different interactions with a triptycene molecule has potential for selective organic transformations and with chiral triptycenes it may advance stereoselective reactions. Until today two chiral triptycene based ligands of type **39** have been synthesized with one of them being used in stereoselective reactions. There seem to be many opportunities remaining with chiral triptycene as ligands or catalysts in organic synthesis including pincer compounds of type **40** (Figure 12).

## Conclusions

Early research on chiral triptycenes was focused to generate various triptycene derivatives using different synthetic routes, to study their absolute configurations and optical properties by correlation methods. A shift from synthetic methodologies to advanced material applications has been observed. Chiral triptycenes have been successfully incorporated into helical polymers, solid supported materials, macrocyclic hosts and as catalysts for stereoselective reactions. Interesting results were obtained when investigating chiral triptycene in polymers, advanced materials and catalysis showing their future potential.

## Conflict of interest

The authors declare no conflict of interest.

## Biographical Information

*Md. Nasim Khan has worked in pharma industries for five years as a research associate in process R&D department. After qualifying GATE and CSIR‐JRF fellowship exams he moved for a PhD degree. He completed his PhD in 2016 at Indian Institute of Technology Patna under the supervision of Dr. Lokman H. Choudhury. His doctoral studies were on the synthesis of “′N′′ & ′′O′′‐heterocycles using multicomponent reaction strategy. After working as an assistant professor in chemistry department at the RK University, he moved to Cardiff University in 2019 to join the group of Prof. Thomas Wirth as a Marie‐Curie postdoctoral fellow, where he is currently doing research in the chemistry of hypervalent iodine reagents*.

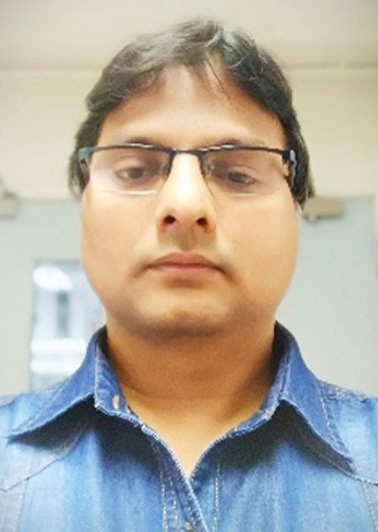

## Biographical Information

*Thomas Wirth is professor of organic chemistry at Cardiff University. After receiving his PhD from TU Berlin, he stayed at Kyoto University as a JSPS fellow. Then he worked independently at the University of Basel before taking up his current position at Cardiff University in 2000. He was awarded the Werner‐Prize from the New Swiss Chemical Society, the Wolfson Research Merit Award from the Royal Society and the Bader‐Award from the Royal Society of Chemistry. In 2016 he was elected as a fellow of The Learned Society of Wales. His main interests of research concern stereoselective electrophilic reactions, oxidative transformations with hypervalent iodine reagents and flow chemistry performed in microreactors*.